# Investigation of the Effects and Mechanisms of *Dendrobium loddigesii* Rolfe Extract on the Treatment of Gout

**DOI:** 10.1155/2020/4367347

**Published:** 2020-09-30

**Authors:** Kai-hui Zhang, Mei-qi Wang, Lu-ling Wei, Cheng-jing Feng, Yu-si Zhang, Jian-bei Teng

**Affiliations:** College of Pharmaceutical Science, Guangxi University of Chinese Medicine, Nanning 530200, China

## Abstract

**Objective:**

Gout is a chronic disease that causes inflammatory arthritis, which is closely related to urate accumulation induced by a disorder of uric acid metabolism and the consequent deposition of monosodium urate crystals. *Dendrobium loddigesii* Rolfe is an herbal medicine that has been used in some traditional Chinese medicine formulae in the treatment of gout. This study aimed to explore and verify the antigout activity of *Dendrobium loddigesii* extract (DLE) on alleviating the hyperuricaemia of mice and the acute gouty arthritis of rats.

**Methods:**

An animal model of hyperuricaemia was established using potassium oxonate (PO). We analysed the expression of uric acid transporter mRNA in the kidney in the hyperuricaemic mice after treatment with DLE. Simultaneously, a monosodium urate crystal-induced acute gouty arthritis rat model was used to evaluate the effects of DLE, according to the level of ankle swelling, as well as the protein levels of inflammatory receptors and cytokines, as assayed by WB and ELISA.

**Results:**

DLE alleviated hyperuricaemia in mice and inhibited acute gouty arthritis in rats (*P* < 0.05). Meanwhile, DLE regulated the levels of uric acid transporters mRNA transcripts, including mouse organic anion transporter 1 (mOAT1), organic anion transporter 3 (mOAT3), urate transporter 1 (mURAT1), and glucose transporter 9 (mGLUT9) in the kidney (*P* < 0.05), suggesting that DLE promoted uric acid metabolism. Furthermore, DLE significantly suppressed the protein levels of TLRs, MyD88, and NF-*κ*B in the ankle joint's synovium (*P* < 0.05), and the serum levels of IL-1*β*, IL-6, and TNF-*α* were also reduced, which demonstrated the anti-inflammatory effects of DLE.

**Conclusion:**

DLE alleviates hyperuricaemia by regulating the transcription level of uric acid transporters in the kidney. It also inhibits acute gouty arthritis by inhibiting the pathway of TLRs/MyD88/NF-*κ*B in the ankle joint's synovium. The findings of the present study imply that DLE alleviates gout by promoting uric acid metabolism and inhibiting inflammation related to the TLRs/MyD88/NF-*κ*B pathway.

## 1. Introduction

Gout is a metabolic disorder that results in hyperuricaemia and the consequent deposition of uric acid crystals in the tissues and joints [1]. The incidence of gout has been increasing for decades [2]. As the monosodium urate (MSU) crystals within the tissues and joints subsequently induce an inflammatory response, the goal of clinical gout treatment is to decrease the serum uric acid (SUA) level and to cure the inflammation [3]. The ultimate cause of the condition, hyperuricaemia, has been associated with overproduction and insufficient excretion of uric acid. Underexcretion of urate has been proven to result in hyperuricaemia [4, 5]. Urate transporters in the kidney, such as urate transporter 1 (URAT1), glucose transporter 9 (GLUT9), urate transporter 1 (OAT1), and organic anion transporter 3 (OAT3), may have essential roles in impaired urate excretion and the resulting hyperuricaemia [6–8]. Thus, they constitute prime targets of agents to treat hyperuricaemia.

Moreover, acute gouty arthritis is an inflammatory situation characterized by red, tender, hot, and swollen joints. Some studies have demonstrated that acute gouty arthritis is associated with the presence of MSU crystals, which serve as a signal affecting specific immune cells, cytokine production, and effector molecule expression, triggering immune responses [9, 10]. Some inflammatory factors, such as interleukin-1*β* (IL-1*β*), interleukin-6 (IL-6), and tumour necrosis factor-*α* (TNF-*α*), have been found to play essential roles in the occurrence and development of acute gouty arthritis [11]. Toll-like receptor 4 (TLR4) is a kind of transmembrane protein receptor that is significantly related to the recognition of MSU crystals in the body. Activation of its downstream signalling molecules, myeloid differentiation factor 88 (MyD88), nuclear factor-*κ*B (NF-*κ*B), and some inflammatory factors can eventually lead to all kinds of inflammatory responses [12]. At the same time, the TLRs/NF-*κ*B signal transduction pathway is one of the main pathways involved in acute gouty arthritis, which can directly regulate the expression of proinflammatory factor IL-1 *β* [13]. These pathways all play extremely notable roles in the regulation of gout-associated immune and inflammatory responses.

There are some antigout drugs available, such as benzbromarone (BBR) and colchicine (COL). They have some adverse effects, such as skin rashes, gastrointestinal disorders, and hepatotoxicity [14–16]. Therefore, the development of new gout treatments that are safe and effective is an urgent unmet need.

Herbal drugs have been used to treat gout in Traditional Chinese Medicine for more than 2000 years [17]. Many studies have been discovering active antigout agents derived from traditional herbal medicines [18–20]. *Dendrobium loddigesii* Rolfe, a perennial epiphytic herb, is widely distributed in the southwestern areas of China, including Guangxi, Yunnan, and Guizhou Provinces [21]. Its stem has been applied to nourish Yin and benefit the stomach, clear away heat-evil, moisten the lungs, and relieve coughs [22]. It has been reported that *Dendrobium loddigesii* has various bioactivities such as anti-inflammatory, antimicrobial, antioxidant, antitumour, and immunomodulatory effects [23–27]. Moreover, we found in previous experiments that the *Dendrobium loddigesii* extract can reduce the level of uric acid in hyperuricaemia mice. However, the mechanism responsible for its antigout action has not been fully elucidated.

In this study, we explored the antigout mechanism of *Dendrobium loddigesii* extract (DLE) based on its effects on hyperuricaemic mice induced by potassium oxonate (PO) and acute gouty arthritis rats induced by monosodium urate (MSU) crystals. The levels of uric acid and creatinine in the mice were measured to explore the effect of DLE on the treatment of hyperuricaemia. We also observed the transcription levels of mOAT1, mOAT3, mURAT1, and mGLUT9 in the kidneys of hyperuricaemic mice after treatment with DLE. Simultaneously, we evaluated the effects of DLE on ankle swelling, protein expression of TLRs/MyD88/NF-*κ*B in the ankle joint's synovium, and the downstream inflammatory factors IL-1*β*, IL-6, and TNF-*α* in the acute gouty arthritis rats.

## 2. Materials and Methods

### 2.1. Animals

Sixty specific pathogen-free (SPF) KM mice, with a body mass of 20 ± 2 g, and 60 specific pathogen-free (SPF) SD rats, with a bodyweight of 200 ± 20 g, were purchased from Hunan SJA Laboratory Animal Co., Ltd. (license number: SCXK (Hunan, China) 2016-0002). All animals were maintained in standard laboratory conditions at 25 ± 2°C, a relative humidity of 50–55%, and a light-dark cycle of 12 hours. All animals were supplied with pure water and standard food. The animals' maintenance and treatment protocols followed the Animal Ethics Committee of Guangxi University of Chinese Medicine (Nanning, China) guidelines for the care and use of laboratory animals. International rules were strictly followed in handling the animals.

### 2.2. Plant Material

*Dendrobium loddigesii* Rolfe was collected from Baise, the Guangxi Zhuang Autonomous Region, China, in August 2018. It was identified by Dr. Hua Zhu (Collaborative Innovation of Zhuang and Yao Ethnic Medicine, Guangxi University of Chinese medicine). It complied with the specification of the Pharmacopoeia of the People's Republic of China (2015).

### 2.3. Preparation of DLE

Based on the experience of drug extraction in our laboratory, the plant was dried at 45°C and powdered in a knife mill. The powder was extracted using ethanol (80%) twice (2 × 120 min) by heat reflux extraction [28]. Then, the continuous filtrate was recovered, and the filter residue was extracted twice in purified water (2 × 120 min) by heat reflux extraction. Then, the two kinds of recovered solutions were mixed. Finally, the recovered solution was subjected to solvent-solvent fractionation to yield the water polarity fraction through extraction by petroleum ether, ethyl acetate, and n-butanol, respectively. The water polarity fraction was concentrated at 65°C in a rotary evaporator and dried on an evaporating dish to obtain the DLE.

### 2.4. Drugs and Reagents

Potassium oxonate (PO) was purchased from Shanghai Macklin Biochemical Co., Ltd. (Shanghai, China). Monosodium urate (MSU) was obtained from Aladdin (Shanghai, China). Benzbromarone (BBR) was purchased from Heumann Pharma (Germany). Colchicine (COL) was purchased from Xishuangbanna Pharmaceutical Co., Ltd. (Yunnan, China). The commercial kits used for determining the levels of UA and Cr were obtained from the Jiancheng Institute of Biotechnology (Nanjing, China). The RevertAid First Strand cDNA Synthesis Kit was purchased from Thermo Fisher Scientific (Shanghai, China). The FastStart Universal SYBR Green Master (Rox) Kit was obtained from Roche (Basel, Switzerland). Enzyme-linked immunosorbent assay (ELISA) kits for the IL-1*β* and IL-6 assays were obtained from Multi Sciences Biotech Co., Ltd. (Hangzhou, China). ELISA kits for TNF-*α* were purchased from Thermo Fisher Scientific (Shanghai, China). Antibodies, including glyceraldehyde-3-phosphate dehydrogenase (GAPDH), toll-like receptor 4 (TLR4), toll-like receptor 2 (TLR2), nuclear factor kappa B p65 (NF-*κ*B p65), and myeloid differentiation factor 88 (MyD88) were purchased from Servicebio Co., Ltd. (Wuhan, China). All other reagents were standard laboratory reagents of analytical grade and were obtained locally.

### 2.5. Drug Dosage

The adult conventional dosage of *Dendrobium loddigesii* was 12.0 g/d. The high (3.0 g/kg/d)/(2.0 g/kg/d), medium (1.5 g/kg/d)/(1.0 g/kg/d), and low (0.75 g/kg/d)/(0.5 g/kg/d) doses used in this study were converted based on the adult dose [29], which were equivalent to twice, equal, and half of the human dose. The positive control drugs used in this study were benzbromarone (BBR) (10 mg/kg/d, 20 mL/kg) and colchicine (COL) (0.3 mg/kg/d, 10 mL/kg), which were equivalent to the adult dose [17, 30, 31].

### 2.6. Models of Hyperuricaemia and Acute Gouty Arthritis and Drug Treatment

#### 2.6.1. Models of Hyperuricaemia and Drug Treatment

Sixty male KM mice were divided into six groups: normal control group, potassium oxonate (PO) model group, PO + benzbromarone (BBR) group, and PO + DLE groups (3.0, 1.5 and 0.75 g/kg/d, 20 mL/kg). Except for the normal control group, PO (250 mg/kg, 10 mL/kg) dissolved in 0.9% saline solution was injected intraperitoneally once a day for seven consecutive days to induce the hyperuricaemic mouse model. Purified water was then given to the normal and model (PO) groups, while the treatment groups got DLE or BBR. The purified water, BBR, and DLE were given intragastrically once daily for six days. After six days of treatment, the animals fasted for 12 h before sacrifice [20, 32].

#### 2.6.2. Models of Acute Gouty Arthritis and Drug Treatment

Sixty SD male rats were divided into six groups: normal control group, monosodium urate (MSU) model group, MSU + colchicine (COL) group, and MSU + DLE groups (2.0, 1.0, and 0.5 g/kg/d, 10 mL/kg). Purified water was given to the normal and model (MSU) groups while the treatment groups got COL or DLE. The purified water, COL, and DLE were fed intragastrically once daily for seven days. On the 5th day, the model was established via a one-time injection of 0.2 mL MSU (25 mg/mL) into the ankle joint cavity 1 h after the drugs were given, and at the same time, the normal control group received an injection of 0.9% saline [33, 34].

### 2.7. Processing of Blood, Urine, and Tissue

Blood and urine samples were collected from the models of hyperuricaemia and acute gouty arthritis to test for the levels of uric acid and creatinine and proinflammatory cytokines, respectively. The kidney from the hyperuricaemia models and the ankle joint's synovium from the acute gouty arthritis models were collected for histological, quantitative real-time polymerase chain reaction (qRT-PCR) and western blot (WB) analysis.

#### 2.7.1. Models of Hyperuricaemia

Blood and urine samples were collected 2 h after the final administration on the 7th day, were allowed to clot for 1 h at room temperature, and were then centrifuged at 3000 rpm for 15 min. These samples were used for the determination of uric acid and creatinine levels, and they were stored at −20°C until the biochemical assays were performed. At the same time, the kidney was cut into pieces for histological and quantitative real-time polymerase chain reaction analysis. Samples for histological analysis were fixed in 4% paraformaldehyde, and samples for qRT-PCR analysis were frozen at −80°C until they were used in the assays.

#### 2.7.2. Models of Acute Gouty Arthritis

Blood samples were collected 48 h after MSU injection, allowed to clot for approximately 1 h at room temperature, and then centrifuged at 3000 rpm for 15 min to obtain the serum. The serum samples were used for the cytokines assay and stored at −20°C until the biochemical tests were performed. Simultaneously, the ankle joint's synovium was collected for histological and western blotting analysis. Samples for histological analysis were fixed in 4% paraformaldehyde and samples for WB analysis were frozen at −80°C until used for the assays.

### 2.8. Levels of Uric Acid and Creatinine in the Mice

The levels of uric acid and creatinine in the serum and urine were assayed by kits (Jiancheng, Nanjing) according to the manufacturer's instructions. The fractional excretion of uric acid (FEUA) was calculated using

(1) $$\begin{array}{c} \text{FEUA}=\frac{\text{serum creatinine }\times \text{ urine uric acid}}{\text{urine creatinine }\times \text{ serum uric acid}}\times 100. \end{array}$$

### 2.9. Analysis of qRT-PCR

Total RNA of the kidney was extracted with TRIzol (Servicebio, Wuhan, China) according to the manufacturer's instructions. Then, the messenger RNAs (mRNAs) were reverse transcribed into cDNAs using the RevertAid First Strand cDNA Synthesis Kit (Thermo Fisher Scientific, Shanghai, China). qRT-PCR was performed to measure the gene expression of OAT1, OAT3, GLUT9, and URAT1. The PCR primers used for RT-PCR were synthesized by Servicebio and the sequences are as follows: OAT1 forward: 5′-CCATACAATCATTCGCACATCC-3′, reverse: 5′-CGTCTGCCGAATCATTGTGG-3′; OAT3 forward: 5′-CAGGCAAACAGGTATGGGTATCA-3′, reverse: 5′-GGTAAGGGCCGATTGAGGGT-3′; GLUT9 forward: 5′-TGGGTCCCTTACCTCAGCATT-3′, reverse: 5′-GCGAAGACGAGGAAGCAGTAG-3′; URAT1 forward: 5′-GAGGAACCAAGCAGGGACAA-3′, reverse: 5′-TGAAGCCAAAGGCAAACCAG-3′; GAPDH forward: 5′-CCTCGTCCCGTAGACAAAATG-3′, reverse: 5′-TGAGGTCAATGAAGGGGTCGT-3′. The RNA samples were normalized to GAPDH, and the relative mRNA levels of the target genes were calculated by the 2^−ΔΔCt^ method.

### 2.10. Analysis of WB

The weighed ankle joint's synovium samples were immersed in precooled RIPA (Servicebio, Wuhan, China) buffer containing phenylmethanesulfonyl fluoride (PMSF) inhibitor and homogenized at 4°C. Then, the homogenate was centrifuged at a speed of 12,000 r/min for 10 minutes and the supernatant was collected. Next, a BCA protein determination kit (Servicebio, Wuhan, China) was used to detect the protein concentration of the sample. These protein samples were mixed with the sample buffer at a ratio of 4 : 1 and boiled for 10 minutes. The proteins were electrophoresed on 10% SDS-PAGE and transferred to PVDF membranes (Millipore, USA) for 1 hour. The membranes were blocked in 5% skim milk powder dissolved in TBST for 2 hours. Then, the membrane was incubated overnight with anti-TLR2 (1 : 1,000), TLR4 (1 : 1,000), NF-*κ*B (1 : 1,000), MyD88 (1 : 1,000), and GAPDH (1 : 1,000) antibodies at 4°C. After washing three times in TBST, we incubated the membrane with a suitable secondary antibody conjugated with HRP (Servicebio, Wuhan, China) for 2 hours at room temperature and then washed the membrane three times in TBST again. The protein bands were detected by an ECL kit (Servicebio, Wuhan, China) and analysed by ImageJ software.

### 2.11. Proinflammatory Cytokines Assay

The levels of IL-1*β*, IL-6, and TNF-*α* in the serum were measured using ELISA kits according to the manufacturer′s instructions.

### 2.12. Assessment of Ankle Swelling

The perimeter of the ankle was measured at 0, 2, 4, 6, 12, 24, and 48 h after MSU crystal injection. They were represented by perimeter (0), perimeter (2), perimeter (4), perimeter (6), perimeter (12), perimeter (24), and perimeter (48). The swelling rate was calculated using

(2) $$\begin{array}{c} \text{swelling rate}=\frac{\text{perimeter}\left(n\right)-\text{perimeter}\left(0\right)}{\text{ perimeter}\left(0\right)}\times 100. \end{array}$$

### 2.13. Analysis of Histology

#### 2.13.1. Models of Hyperuricaemia

The mouse kidneys were fixed for 24 h at room temperature in 4% paraformaldehyde solution. Paraffin sections 4 *μ*m thick were prepared from each kidney, then stained with haematoxylin and eosin (H&E), and inspected under a light microscope (DS-Ri2, Nikon Instruments Inc., Japan) at *a* × 400 magnification. A histopathological score was applied in a blinded fashion [35]. A score was given for tubular necrosis, tubule dilatation, loss of the brush border, and dilatation of Bowman's capsule for 10 randomly chosen fields of view for each slide at *a* × 200 magnification. The score was as follows: 0 (none), 1 (≤10%), 2 (11–25%), 3 (26–45%), 4 (46–75%), and 5 (≥76%).

#### 2.13.2. Models of Acute Gouty Arthritis

The ankle joints of MSU-induced gouty arthritis rats were fixed for 24 h at room temperature in 4% paraformaldehyde solution. Then, they were decalcified in EDTA solution, embedded in paraffin and sectioned at 5 *µ*m, stained with H&E, and examined under a light microscope at ×100 magnification.

### 2.14. Statistical Analysis

In this experiment, SPSS 21.0 software was used for data processing. The graphs were drawn with GraphPad Prism 7.0. All data were subjected to one-way analysis of variance (ANOVA) and expressed by mean ± standard deviation (SD). LSD *t*-tests were applied when the homogeneity of variance assumptions was satisfied; otherwise, Dunnett's *t*-test was used. *P* < 0.05 was statistically significant.

## 3. Results and Discussion

### 3.1. Results

#### 3.1.1. DLE Affects the Levels of UA, Cr, and FEUA in Hyperuricaemic Mice

**Figure 1:**
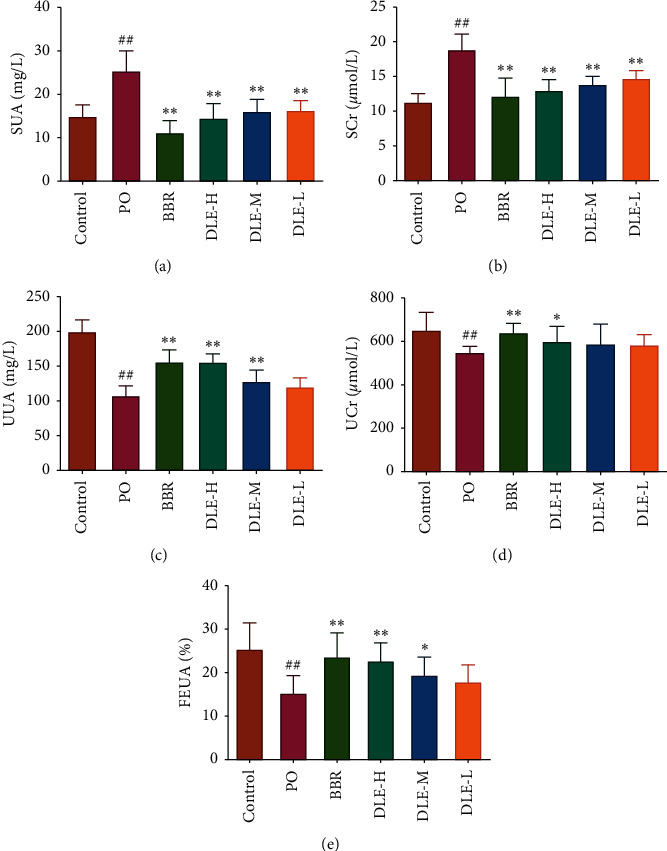
The effects of DLE on the levels of UA (a and c) and Cr (b and d), as well as FEUA (e) in hyperuricaemic mice. DLE-H: high dose of DLE (3.0 g/kg body weight); DLE-M: medium dose of DLE (1.5 g/kg body weight); DLE-L: low dose of DLE (0.75 g/kg body weight). ^##^*P* < 0.01 compared with the control group; ^##^*P* < 0.01, ^*∗*^*P* < 0.05 compared with the PO group (*n* = 10).

As shown in Figure 1, after being intraperitoneally administered with potassium oxonate (PO) for seven consecutive days, the level of serum uric acid (SUA) in the model control group was significantly higher than that in the normal control group (*P* < 0.01), which indicated that the model of hyperuricaemia was successfully established. Compared with the model (PO) group, the level of SUA was suppressed significantly (*P* < 0.01). In contrast, the level of urine uric acid (UUA) was increased dramatically in response to DLE in a dose-dependent manner (the high and medium doses had a significant difference, *P* < 0.01). The serum creatinine (SCr) level can be associated with renal dysfunction. Renal damage can be accompanied by an increase in SCr, indicating reduced creatinine clearance. Compared with the model (PO) group, the levels of SCr were suppressed significantly by DLE. Moreover, DLE treatment at the high and medium doses could substantially increase the renal UA handling ability of FEUA (*P* < 0.01 and *P* < 0.05, respectively) in a dose-dependent manner and enhance UA excretion. It follows that DLE alleviated the hyperuricaemia, and this effect inhibited the deposition of UA.

#### 3.1.2. Effects of DLE on Renal Histopathology in Hyperuricaemic Mice

**Figure 2:**
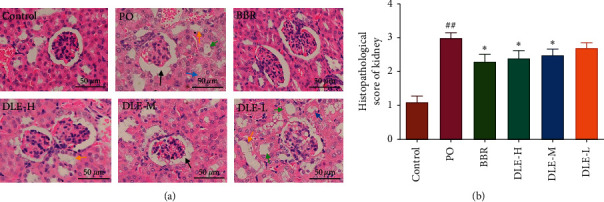
Histology of the kidney in hyperuricaemic mice (a), H&E (×400), and histopathological score of the kidney (b). Dilatation of Bowman's capsule (black arrows), loss of brush border (green arrows), tubule dilatation (yellow arrows), and tubular necrosis (blue arrows). DLE-H: high dose of DLE (3.0 g/kg body weight); DLE-M: medium dose of DLE (1.5 g/kg body weight); DLE-L: low dose of DLE (0.75 g/kg body weight). ^##^*P* < 0.01 compared with the control group; ^*∗*^*P* < 0.01 compared with the PO group (*n* = 10).

According to the results of the serum biochemistry analysis, the level of SUA could be restored by all doses of DLE. Our histological findings support the observations of UA level changes after treatment and are consistent with the SUA observations in the hyperuricaemic mice model. As shown in Figure 2, renal sections from the normal control group showed typical renal architecture. Compared with the normal mice, the model (PO) group showed apparent pathological changes (*P* < 0.01). Compared with the model (PO) group, the high and medium doses of DLE significantly reduced the degree of kidney damage (*P* < 0.05). The trend of the histopathological score of the kidney in each group is consistent with the level in SCr. This shows that DLE improved renal function and promoted UA excretion.

#### 3.1.3. Effects of DLE on the Transcription Levels of mOAT1, mOAT3, mURAT1, and mGLUT9 in the Kidney

**Figure 3:**
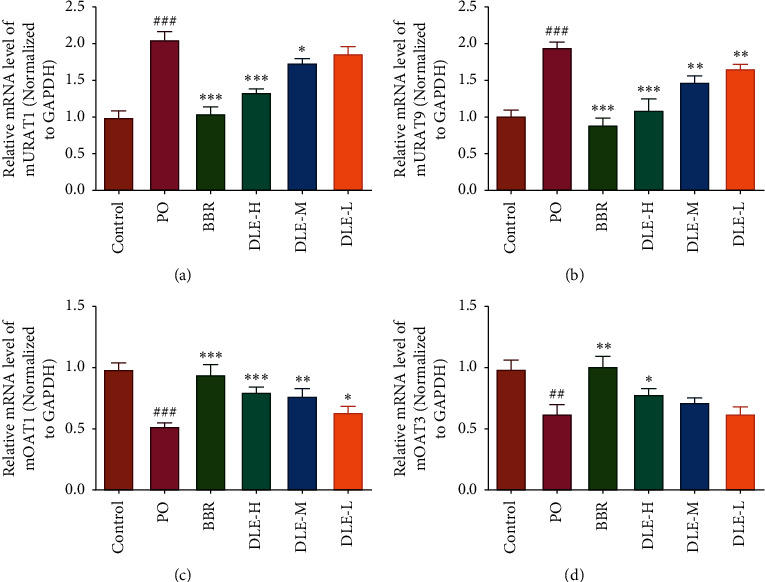
The effects of DLE on the transcription levels of mURAT1 (a), mGLUT9 (b), mOAT1 (c), and mOAT3 (d) in renal tissues in hyperuricaemic mice. DLE-H: high dose of DLE (3.0 g/kg body weight); DLE-M: medium dose of DLE (1.5 g/kg body weight); DLE-L: low dose of DLE (0.75 g/kg body weight). ^###^*P* < 0.001 and ^##^*P* < 0.01 compared with the control group; ^*∗∗∗*^*P* < 0.001, ^*∗∗*^*P* < 0.01, and ^*∗*^*P* < 0.05 compared with the PO group (*n* = 10).

As shown in Figure 3, compared with the normal control group, PO administration induced a significant downregulation of the transcription levels of mOAT1 and mOAT3 in mice kidney (*P* < 0.001 and *P* < 0.01), as well as a substantial upregulation of mURATl and mGLUT9 (*P* < 0.001). The high and medium doses of DLE significantly decreased the mURATl transcription level in a dose-dependent manner (*P* < 0.001 and *P* < 0.05) compared with the model (PO) group, as shown in Figure 3(a). As shown in Figure 3(b), DLE reduced the transcription level of renal mGLUT9 in a dose-dependent manner (*P* < 0.001 and *P* < 0.01). As shown in Figure 3(c), DLE increased the transcription level of renal mOAT1 in a dose-dependent fashion (*P* < 0.001, *P* < 0.01, and *P* < 0.05). However, as shown in Figure 3(d), only the high dosage of DLE increased the transcription level of renal mOAT3 (*P* < 0.05). Thus, it can be seen that DLE regulates UA metabolism, including reabsorption and excretion, by regulating uric acid transporters.

#### 3.1.4. Effects of DLE on the Histopathology of the Ankle Joint's Synovium in Acute Gouty Arthritis Rats

**Figure 4:**
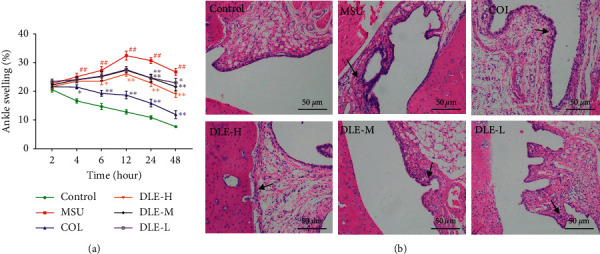
Change of ankle swelling (a). Histology of ankle joint's synovium in acute gouty arthritis rats (b), H&E (×100). Infiltration of inflammatory cells (black arrows). DLE-H: high dose of DLE (2.0 g/kg body weight); DLE-M: medium dose of DLE (1.0 g/kg body weight); DLE-L: low dose of DLE (0.5 g/kg body weight) (*n* = 10).

As shown in Figure 4(a), we investigated the mechanism of DLE in alleviating the ankle swelling of rats after the injection of the MSU crystal suspension. Treatment with DLE was able to reduce paw oedema significantly at any tested dose. As shown in Figure 4(b), the acute gouty arthritis rats had apparent synovial hyperplasia and infiltration of inflammatory cells in the synovium. The colchicine (COL) treatment partially reduced the inflammatory cell infiltration. At the same time, the DLE treatment dose-dependently improved the synovial hyperplasia and reduced the inflammatory cell infiltration into the synovium. This shows that DLE reduced joint injury.

#### 3.1.5. Effects of DLE on the Levels of IL-1*β*, IL-6, and TNF-*α* in MSU Crystal-Induced Rats

**Figure 5:**
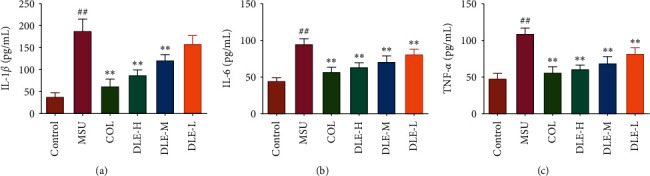
The effects of DLE on IL-1*β* (a), IL-6 (b), and TNF-*α* (c) in acute gouty arthritis rats. DLE-H: high dose of DLE (2.0 g/kg body weight); DLE-M: medium dose of DLE (1.0 g/kg body weight); DLE-L: low dose of DLE (0.5 g/kg body weight). ^##^*P* < 0.01 compared with the control group; ^*∗∗*^*P* < 0.01 compared with the MSU group (*n* = 10).

The levels of three proinflammatory cytokines in the serum samples were determined as shown in Figure 5(a)∼5(c). The MSU crystals induced a significant increase of IL-1*β*, IL-6, and TNF-*α* levels in the serum, and compared with the model (MSU) group, treatment with high and medium doses of DLE significantly reduced the production of IL-1*β* and all doses of DLE reduced IL-6 and TNF-*α* towards the normal level. The positive control drug COL also considerably decreased the levels of the proinflammatory cytokines compared with the model (MSU) group. Since the serum inflammatory factors were reduced by DLE, this indicated that DLE has anti-inflammatory functions.

#### 3.1.6. Effects of DLE on TLR2, TLR4, MyD88, and NF-*κ*B Protein Levels in Rat Ankle Joint's Synovium

**Figure 6:**
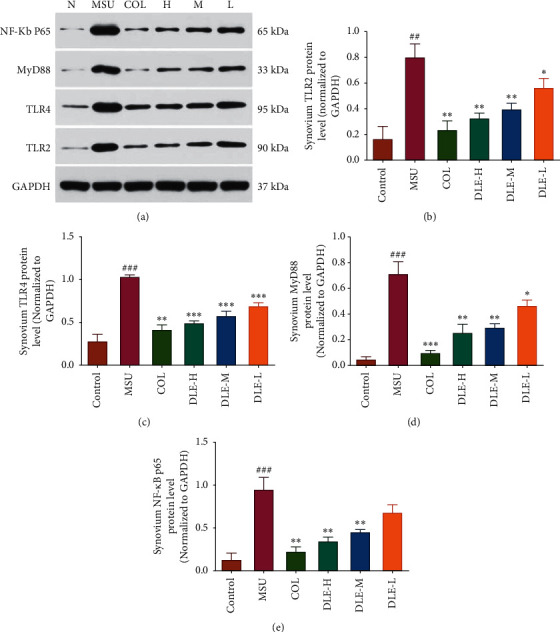
(a) The effects of DLE on TLR2 (b), TLR4 (c), MyD88 (d), and NF-*κ*B (e) protein levels in rat ankle joint's synovium. DLE-H: high dose of DLE (2.0 g/kg body weight); DLE-M: medium dose of DLE (1.0 g/kg body weight); DLE-L: low dose of DLE (0.5 g/kg body weight). ^###^*P* < 0.001 and ^##^*P* < 0.01 compared with the control group; ^*∗∗∗*^*P* < 0.001, ^*∗∗*^*P* < 0.01, and ^*∗*^*P* < 0.05 compared with the MSU group (*n* = 10).

As shown in Figure 6, the protein levels of TLR2, TLR4, MyD88, and NF-*κ*B in the model (MSU) group were significantly increased (*P* < 0.01 and *P* < 0.001) compared with the normal control group. All doses of DLE significantly decreased the TLR2 and MyD88 protein levels in a dose-dependent manner (*P* < 0.01, *P* < 0.01, and *P* < 0.05, respectively) compared with the model (MSU) group, as shown in Figures 6(b) and 6(d). As shown in Figure 6(c), compared with the model (MSU) group, all doses of DLE significantly decreased the TLR4 protein level (*P* < 0.001). Compared with the model (MSU) group, the DLE treatment in the high and medium groups significantly reduced the expression of NF-*κ*B in the ankle joint's synovium, as shown in Figure 6(e). Therefore, it is inferred that DLE regulates the TLRs/MyD88/NF-*κ*B pathway, which results in anti-inflammatory effects.

## 4. Discussion

Gout is a treatable and common form of inflammatory arthritis. The central pathological feature of gout is the chronic deposition of MSU crystals, which form when the concentration of UA increases [36–38]. In these years, along with the improvements in living standards, the incidence of hyperuricaemia and gout has gradually increased [39]. The number of drugs used in clinical practice for treating hyperuricaemia or gout is limited, and there are numerous side effects associated with them. Therefore, the development of safer and more active agents, especially herbal medicine, is urgently needed. Potassium oxonate is widely used to induce hyperuricaemic mice [40]. In this study, hyperuricaemia was induced with 250 mg/kg of potassium oxonate. Compared with the normal control group, their SUA level was increased significantly, indicating that the model of hyperuricaemia was built successfully. In this study, we appraised the effect of DLE on the levels of UA and Cr in the hyperuricaemic mice. qRT-PCR was used to observe the transcription levels of mOAT1, mOAT3, mURAT1, and mGLUT9 in hyperuricaemic mice after treatment with DLE. At the same time, we evaluated the effects of DLE on ankle swelling, protein expression of TLRs/MyD88/NF-*κ*B, and downstream inflammatory factors IL-1*β*, IL-6, and TNF-*α* in acute gouty arthritis rats.

Serum creatinine levels can be associated with renal dysfunction. Renal damage can be accompanied by an increase in serum creatinine, indicating reduced creatinine clearance [41]. Compared with the model (PO) group, the level of serum creatinine (SCr) was suppressed significantly (*P* < 0.01). Moreover, the DLE at high and medium doses could dramatically increase the renal UA handling parameter FEUA (*P* < 0.01 and *P* < 0.05 respectively) in a dose-dependent manner and enhance UA excretion. Simultaneously, the histopathology results revealed that DLE significantly reduced the kidney damage in the hyperuricaemic mice. The data in general support the effects of DLE on reducing the level of serum uric acid.

To further explore the mechanisms of action involved, the qRT-PCR data in Figure 3 show that DLE possesses a potent uricosuric effect in hyperuricaemic mice through regulating renal mURAT1, mGLUT9, and mOAT1, which results in an enhancement of UA excretion. DLE also has the potential to control renal mOAT3 at a high dose (*P* < 0.05). It has been reported that GLUT9 and URAT1 are the primary mediators of urate reabsorption in the tubular system in mice and humans, while OAT1 and OAT3 have the function of urate excretion [42–44]. The data from these observations provide mechanistic information on DLE in the treatment of hyperuricaemia.

Acute gouty arthritis is an inflammatory response sparked by the abnormal metabolism of UA, leading to the deposition of MSU crystals in the joints, causing neutrophil infiltration and consequently swelling and pain [45, 46]. Therefore, the treatment of inflammation is the ideal therapeutic approach against acute gouty arthritis. The rat model we used here is a well-established model of acute gouty arthritis, whose typical symptom is ankle swelling observed in the last 24 hours after MSU crystals injection into the joint space. DLE showed a significant reduction in ankle oedema compared with the model (MSU) group. In addition, the histopathology results revealed that DLE dramatically reduced neutrophil infiltration into the ankle joint's synovium. These findings suggested that DLE could protect rats from MSU crystal-induced acute gouty arthritis.

Colchicine is currently used as first-line therapy for inflammation in acute gouty arthritis. It also reduces the level of proinflammatory cytokines, including IL-1*β*, IL-6, and TNF-*α*, which contributes to the improvement of the inflammatory responses induced by MSU crystals [47]. Similar to colchicine, DLE may serve as an active agent against acute gouty arthritis mediated by inhibition of proinflammatory cytokines' secretion. Moreover, the effect of DLE on the pathogenesis of acute gouty arthritis was further investigated. NF-*κ*B signalling plays a central role in numerous inflammation-related diseases, such as acute gouty arthritis. The TLR pathway could regulate it, and a MyD88-dependent signal is an essential key to the NF-*κ*B signals [48]. We observed that the expression levels of TLR2, TLR4, MyD88, and NF-*κ*B p65 were dramatically enhanced in MSU-stimulated ankle joint's synovium in acute gouty arthritis rats. DLE intervention significantly reduced the mediators listed above, which indicated that the mechanism of DLE for anti-inflammation was related to the TLRs/MyD88/NF-*κ*B pathway.

In this study, it was observed that DLE showed a significant reduction in ankle oedema induced by MSU crystals. DLE also increased urinary excretion of uric acid, indicating a synergistic action that may have efficacy in the treatment of hyperuricaemia and acute gouty arthritis.

## 5. Conclusions

In conclusion, DLE exhibited remarkable activity in the treatment of gout. It possesses a potent uricosuric effect in hyperuricaemia mice through regulating the expression of renal uric acid transporters, including mGLUT9, mURAT1, and mOAT1, which contributes to the enhancement of uric acid excretion and the protection of the kidney from hyperuricaemia-induced dysfunction. Meanwhile, DLE significantly alleviated the ankle swelling of MSU crystal-induced acute gouty arthritis in rats, achieving an anti-inflammatory effect by downregulating the protein expression of TLRs/MyD88/NF-*κ*B and their downstream inflammatory factors IL-1*β*, IL-6, and TNF-*α*. This study for the first time explored the antigout activity of an extract of *Dendrobium loddigesii* in hyperuricaemic mice and acute gouty arthritis rats.

## Data Availability

The data used to support the findings of this study are included in the article and supplementary materials.

## Supplementary Materials

Supplementary Materials Figure S1: changes in the weight of mice. The green line indicates the weight of the control group, and the red line shows the weight of the DLE group.
