# Supplementary material for: Investigation of the Effects and Mechanisms of Dendrobium loddigesii Rolfe Extract on the Treatment of Gout
Source: Evid Based Complement Alternat Med. 2020 Sep 30;2020:4367347. doi: 10.1155/2020/4367347 (PMC7547349; doi:10.1155/2020/4367347)
Supplement: Supplementary Materials — Figure S1: changes in the weight of mice. The green line indicates the weight of the control group, and the red line shows the weight of the DLE group. [file 4367347.f1.pdf]

Supplementary Material

1. Drug toxicity analysis

1.1. Study on maximal tolerable dose (MTD) of DLE in mice

Since DLE is less toxic, the median lethal dose (LD50) couldn't be measured in our study. So we assessed the acute toxicity of the drug through MTD by observing the acute poisoning reaction and death of KM mice after three times of intragastric administration of the maximum dose of DLE within 24 hours.

1.1.1. Grouping and administration

40 specific pathogen-free (SPF) KM mice, female and male. KM mice were divided into two groups: the control group and DLE group (240g/kg/d), 20 per group, half male and half female. DLE group was administrated intragastrically DLE, 2 g/mL(The maximum concentration administrated intragastrically that can be configured under our laboratory conditions), 40 mL/kg. The Control group was given an equal volume of pure water. Three times of intragastric administration at intervals of 6-8 hours within 24 hours.

1.1.2. Observation, record, and research

After administration, observe continuously for 6 hours after administration and then observe twice a day for 14 days and record body weight. On day 15, the surviving mice were killed for pathological examination.

1.1.3. Results of clinical observation and weight change

No symptoms of poisoning and no deaths were found after continuous observation for 6 hours after administration in the DLE group. Under observing continuously for 14 days after administration, the mice in each group were in good mental state, having normal behaviors, with brighter coats, and there were no abnormal secretions, redness, swelling, and ulceration in the auricles, eyes, snout, and perineum. All mice were surviving. As shown in Figure S1, It can be seen that DLE has no significant effect on the weight of mice.

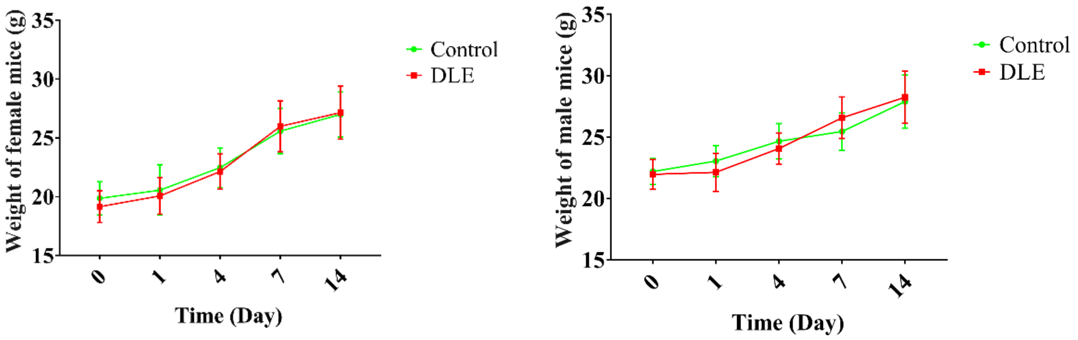

Figure S1: Changes in the weight of mice, green line indicated the weight of the control group, and the red line showed the weight of the DLE group.

1.1.4. Results of pathological examination.

After pathological dissection, no abnormalities or lesions were found in the heart, liver, spleen, lung, kidney, stomach, and intestines by gross examination. As shown in Figure S2, there was no significant difference in the liver pathology section between the control group and the DLE group.

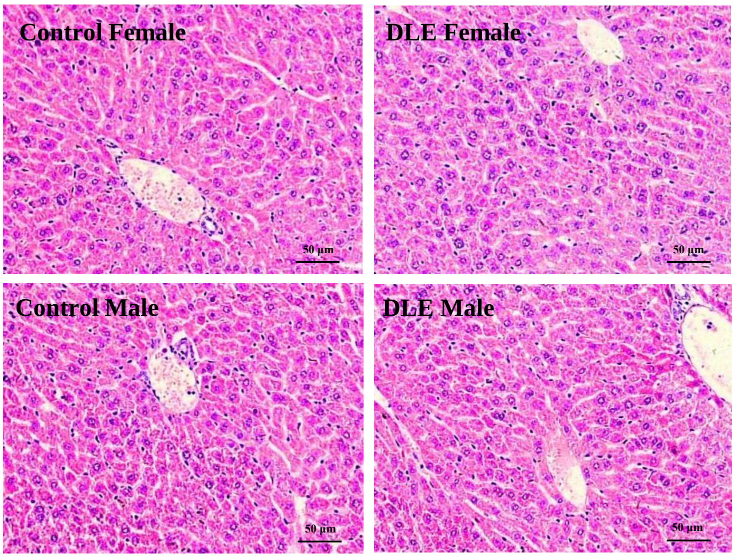

Figure S2: Histology of the liver in control and DLE group. H & E Stain (×200)

1.1.5. The MTD of DLE in mice

After giving the maximum dose of DLE, there were no signs of toxicity and no death in the DLE group. Therefore, the MTD of DLE in mice was at least 240g/kg/d. According to the clinical dose for adults (60kg), 12g/d, it is equivalent to 1200 times the oral dose of clinical adults one day.
